# Comprehensive assessment of myocardial strain in post-infarct mice using 3D Cine DENSE

**DOI:** 10.1186/1532-429X-11-S1-P149

**Published:** 2009-01-28

**Authors:** Xiaodong Zhong, Brent A French, Craig H Meyer, Christopher M Kramer, Frederick H Epstein

**Affiliations:** University of Virginia, Charlottesville, VA USA

**Keywords:** Remote Region, Post Myocardial Infarction, Infarct Region, Myocardial Strain, Cardiac Contractile Function

## Introduction

Transgenic and knockout mice are widely used to study the roles of individual genes in cardiovascular disease. Quantitative MR imaging methods for assessing 3D strain at high resolution in mice are needed to elucidate the roles of individual genes in modulating cardiac contractile function.

## Purpose

To develop 3D cine DENSE (Displacement Encoding with Stimulated Echoes) MRI for comprehensive myocardial strain imaging of the mouse heart.

## Methods

An ECG-gated segmented 3D spiral cine DENSE pulse sequence with online image reconstruction was implemented on a 7 T MRI scanner (Bruker Clinscan, Germany). A stack of spirals *k*-space trajectory was employed for 3D spatial encoding, rapid data acquisition, and short echo time. Three-point phase cycling was used for artifact suppression, 4-point displacement encoding was used to efficiently measure 3D motion, and a low resolution field map was acquired for online spiral deblurring. To further ensure minimal blurring, the spiral readout duration was limited to 3 ms. In accordance with protocols approved by the animal care and use committee at our university, 3 mice were imaged before and 1 day after experimental myocardial infarction (MI). During MRI, mice were anesthetized with isoflurane and maintained at 37°. ECG and respiration were monitored using an MRI-compatible system for small animals (SAII, Stony Brook, NY). Specific pulse sequence parameters included TR = 6 ms, TE = 1 ms, matrix = 128 × 128 × 8, and displacement-encoding frequency = 2.5 radians/mm. The field of view was 32 × 32 × 3.2 mm^3^, which covered the entire middle section of the mouse left ventricle (the longitudinal dimension of the mouse left ventricle is approximately 6 mm). These parameters provided voxel size = 0.25 × 0.25 × 0.4 mm^3^, temporal resolution = 6 ms, typically 18 cardiac phases across the cardiac cycle, and a scan time of approximately 45 minutes, depending on heart and respiratory rates. Additionally, on day-1 post-MI, delayed Gd-DTPA-enhanced inversion-recovery MRI was performed to image the region of infarction. For quantitative analysis of DENSE data, images were exported to a PC and manually segmented. Tissue tracking and strain analysis were performed using 3D extensions of 2D methods that were described previously.

## Results

**Figure 1:**
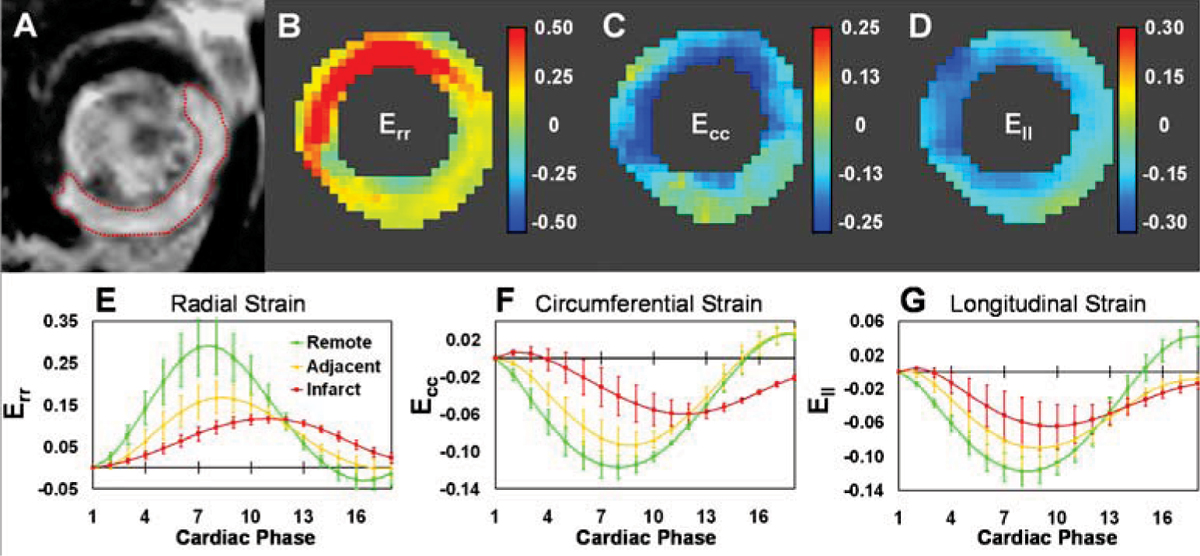
Figure 1

Example images and strain maps are shown in Figure 1 for a mouse 1 day after MI. Specifically, the Gd-enhanced image (Panel A) defined the infarcted, adjacent (within 1 mm of infarction) and remote regions. Panels B – D show the corresponding end-systolic radial (E_rr_), circumferential (E_cc_) and longitudinal (E_ll_) strain maps for the same slice. Abnormal strain generation is evident in the infarcted and adjacent regions of each map. Panels E through G show strain-time curves (mean ± SEM) from the mid-ventricle for the infarct, adjacent, and remote regions, where severely reduced strain was measured in the infarct region, intermediate strain characterized the adjacent region, and nearly normal strain was found in the remote region. In the day-1 post MI infarct region, the times to peak strain were also all significantly delayed. In contrast to data from post-MI mice, baseline data showed normal strains throughout the mid ventricle.

## Conclusion

Cine DENSE methods were developed that provide a quantitative assessment of 3D strain throughout the mid-section of the mouse LV in a scan time of approximately 45 minutes. Using the parameters provided here, DENSE images had high signal-to-noise ratio and high temporal and spatial resolutions. The high-quality displacement images lead to high resolution 3D strain data that clearly distinguished differences in function between infarcted, adjacent, and remote regions in post-infarct mice. When applied to genetically-engineered mice, these accurate high resolution data may help elucidate the roles of individual genes in modulating post-infarct regional function and LV remodeling.

